# Trained Immunity of IL-12-, IL-15-, and IL-18-Induced CD_3_+CD_56_+ NKT-Like Cells

**DOI:** 10.1155/2022/8724933

**Published:** 2022-06-23

**Authors:** Siyu Zhu, Chen Zhang, Qian Sun, Yang Wang, Wenwen Yu, Feng Wei, Xiubao Ren

**Affiliations:** ^1^Department of Immunology and Biotherapy, Key Laboratory of Cancer Immunology and Biotherapy, Tianjin Medical University Cancer Institute and Hospital, National Clinical Research Center for Cancer, Key Laboratory of Cancer Prevention and Therapy, Tianjin's Clinical Research Center for Cancer, Tianjin 300060, China; ^2^Department of Minimally Invasive Esophageal Surgery, Key Laboratory of Cancer Prevention and Therapy, National Clinical Research Center for Cancer, Tianjin Medical University Cancer Institute and Hospital, Tianjin, China

## Abstract

CD_3_+CD_56_+ natural killer T (NKT)-like cells have an immune function of T cells and NK cells, which play an important role in antitumor and antiviral immune responses. This study aims to establish a CD_3_+CD_56_+ NKT-like cell model by simulating the memory NK effect induced by cytokines IL-12, IL-15, and IL-18 (IL-12/15/18) and explore the formation mechanism. Our study found that the IL-12/15/18 preactivated CD_3_+CD_56_+ NKT-like cells exhibited enhanced IFN-*γ* production in response to restimulation with IL-12/15/18 for 6h on day 7. The intrinsic potential of these trained cells was significantly improved, showing an increase in IFN-*γ*, TNF-*α*, and cell proliferation potential. The IFN-*γ* release, granzyme B level, and proliferation ability significantly increased when stimulated by NK-cell-sensitive K562 tumor cells. Among these cytokines, the combination of IL-12/15/18 was particularly effective. After the preactivation of IL-12/15/18, some cell surface proteins related to function and differentiation, such as CD11b, CD62 L, NKp46, NKG2A, and CD127, showed an evident and consistent change trend. The CDK4/6 inhibitor can effectively weaken this effect, and the expression of cyclin D1, Rb protein phosphorylation, and E2F-1 decreased significantly. Our work revealed that cytokine IL-12/15/18 can induce CD_3_+CD_56_+ NKT-like cells to obtain enhanced training immunity, which was a memory-like phenomenon.

## 1. Introduction

Sharing characteristics with natural killer (NK) and T cells, CD_3_+CD_56_+ natural killer T (NKT)-like cells possess innate and acquire immune functions [1]. They regulate different immune responses, including cancer immunity and autoimmunity development [1, 2]. NK cells, iNKT cells, and CD_3_+CD_56_+ NKT-like cells serve as the first line of defense of the natural immune system [3]. These cells play an important role in inhibiting viral infection and controlling tumor by secreting interleukin, interferon (IFN), and chemokine [4, 5]. CD_3_+CD_56_+ NKT-like cells are large granular lymphocytes, which are CD1 d unrestricted, and they have polygenic TCR recombination [1, 6]. Moreover, they can be activated to secrete cytokines and effectively kill cancer cells in a nonmajor histocompatibility complex (MHC)-restricted lysis in the absence of T-cell antigen receptor (TCR) activation [1–4]. Numerous studies have confirmed that exogenous IFN-*γ* and cytokines such as IL-2 and IL-15 can activate immune cells [5]. As far as we know, the nature, function, and clinical relevance of cytokine-trained CD_3_+CD_56_+ NKT-like cells remain unexplored.

Immunotherapy is a potentially valuable treatment of the 21st century [6]. Relevant immunotherapies in recent clinical trials engage either the cellular stage of adaptive immunity or effector molecules, such as cytokines. Trained immunity was first proposed by Netea et al. in recent years [7, 8]. As an immunological memory of the innate immune system, trained immunity involves the epigenetic programming of myeloid lineage cells, leading to changes in their metabolic and phenotypical behavior [6, 9]. The above-mentioned process may strengthen the immune response to secondary stimuli [9–11]. Cooper MA first proposed cytokine-induced memory-like NK cells in 2009, representing a typical example of trained immunity of NK cells [12]. Since then, an increasing number of studies have confirmed that human NK cells display functional memory-like properties after cytokine activation, which may provide a novel rationale for NK cell immunotherapy strategies [4, 13]. Therefore, we investigate whether CD_3_+CD_56_+ NKT-like cells can establish a similar cytokine-induced memory NK cell experimental model when they play a role in NK cell-killing activity.

This study aimed to establish a CD_3_+CD_56_+ NKT-like cell model by simulating the memory NK effect induced by cytokines. The levels and functional activities of CD_3_+CD_56_+ NKT-like cells in human peripheral blood mononuclear cells (PBMC) stimulated by IL-12, IL-15, and IL-18 (IL-12/15/18) were detected to observe their response to NK-cell-sensitive K562 tumor cells. The biological activity and function of IL-12/15/18 preactivated CD_3_+CD_56_+ NKT-like cells were analyzed when the same cytokines were restimulated. Moreover, the mechanism of this memory-like NK effect produced by CD_3_+CD_56_+ NKT-like cells was explored.

## 2. Materials and Methods

### 2.1. Blood Samples

Fresh human blood specimens for flow cytometry experiments were obtained from Tianjin Medical University Cancer Hospital. All samples were provided anonymously after informed consent was obtained. The collection, distribution, and usage of all deidentified human peripheral blood were approved by the Institutional Review Committee on Human Research of the Tianjin Medical University Cancer Institute and Hospital.

### 2.2. Cell Lines and Culturing Conditions

Human leukemia cell lines (K562) were obtained from the American Type Culture Collection. All cancer cell lines were maintained in RPMI 1640 media (Gibco, Grand Island, New York, NY, USA) supplemented with 10% heat-inactivated fetal bovine serum (Thermo Fisher Scientific) and contained 100 U/mL of penicillin and 100 *µ*g/mL of streptomycin (Gibco).

### 2.3. Cell Culture

PBMCs from healthy adult peripheral blood samples were isolated by Ficoll centrifugation. Human PBMCs were plated at 3–5 × 10^6^ cells/mL and preactivated overnight (about 16 h) using rhIL-12 (10 ng/mL, PeproTech) + rhIL-18 (50 ng/mL, MBL International) + rhIL-15 (1 ng/mL, PeproTech) or control (rhIL-15, 1 ng/mL) conditions, washed three times to remove cytokines, and then added with rhIL-15 (1 ng/mL) to maintain the culture in a complete RPMI 1640 medium containing 10% fetal bovine serum. After a week of culture at 37°C in a 5% CO_2_ incubator, the cells were washed, added with PMA + Ionomycin (cell activation cocktail with brefeldin was obtained from BioLegend (San Diego, CA), the same set of cytokines IL-12/15/18, K562 tumor cell lines, 0.1–1 *µ*M of the CDK4/6 inhibitor, Myc inhibitor, DNA-demethylating agent, and enhancer of zeste homolog2 (EZH2) inhibitor separately for 5 h), and then washed three times with phosphate-buffered saline.

### 2.4. Flow Cytometry and Antibodies

Cells were harvested and transferred into a 96-well plate at approximately 1 × 10^6^/well for staining, incubated with human Fc receptor blocking solution (422302; BioLegend), and then stained in sequence with cell viability dyes (423106; BioLegend), surface antibodies, and intracellular antibodies. The release of IFN-*γ*, TNF-*α*, and granzyme B, proliferation, and degranulation were detected by flow cytometry. Flow cytometry antibodies, including anti-CD56 (HCD56, 318318), anti-CD3 (UCHT1, 300447), anti-IFN-*γ* (B27, 506529), anti-Ki67 (Ki-67, 350519), anti-TNF-*α* (Mab11, 502915), anti-Granzyme B (QA16A02, 372207), CD62 L (DREG-56,304823), CD127 (A019D5, 351333), NKp46 (9E2, 331931), CD11b (ICRF44, 301305), CD27 (O323, 302805), CD45Ra (HI100, 304105), and CD57 (HCD57, 322316), were obtained from BioLegend (San Diego, CA). Anti-NKG2A (REA110, 130-113-563) was obtained from Miltenyi Biotec (Bergisch Gladbach, Germany). CDK4/6 inhibitor (HY-16297A, MCE), Myc inhibitor (HY-13865, MCE), DNA-demethylating agent (HY-A0004, MCE), and EZH2 (HY-15555, MCE) were obtained from Selleckchem.com. Data were acquired using a LSRFortessa flow cytometer (BD Biosciences).

### 2.5. Western Blot

All harvested cells were lysed in ice-cold RIPA lysis buffer (Beijing Solarbio Science & Technology Co., Ltd.). All protein concentrations were determined using the BCA protein assay reagent and measured with a NanoDrop2000 spectrophotometer (Thermo Fisher Scientific, Inc.). Equal amounts of protein samples (25 µg) were separated by 10% SDS-PAGE gels and then transferred onto PVDF membranes. After blocking with nonfat milk for 2 h at room temperature, the membranes were probed with the corresponding primary antibodies incubated at 4°C overnight. After incubating with horseradish peroxidase (HRP)-conjugated secondary antibodies at room temperature for 2 h, protein bands were visualized using a chemiluminescence detection system (Bio-Rad Laboratories, Inc.). Then, the density was evaluated by ImageJ 1.48v (National Institutes of Health). Primary antibody diluent areas are as follows: CDK4 (cat. no. 12790; Cell Signaling Technology, Inc.), CDK6 (cat. no. 13331; Cell Signaling Technology, Inc.), Cyclin D1 (cat. no. 55506; Cell Signaling Technology, Inc.), Phospho-Rb (cat. no. 3590; Cell Signaling Technology, Inc.), E2F-1 (cat. no. 3742; Cell Signaling Technology, Inc.), GAPDH (cat. no. 5174; Cell Signaling Technology, Inc.), and Histone H3 (cat. no.4499; Cell Signaling Technology, Inc.). All the aforementioned antibodies were used at 11000 except for H3(12000). HRP-conjugated antirabbit (cat. no. SA00001-2; 1:10000; Proteintech Group, Inc.) and antimouse (cat. no. SA00001-1; 110000; Proteintech Group, Inc.) secondary antibodies were used.

### 2.6. Statistical Analysis

Data are shown as the meansSEM in all graphs, and significant differences were calculated with Student's *t*-test or as indicated. *P* < 0.05 was considered significant. All statistical analyses and plots were produced in GraphPad Prism.

## 3. Results

### 3.1. IL-12/15/18-Induced CD_3_+CD_56_+ NKT-Like Cells Increase IFN-*γ* Release

**Figure 1:**
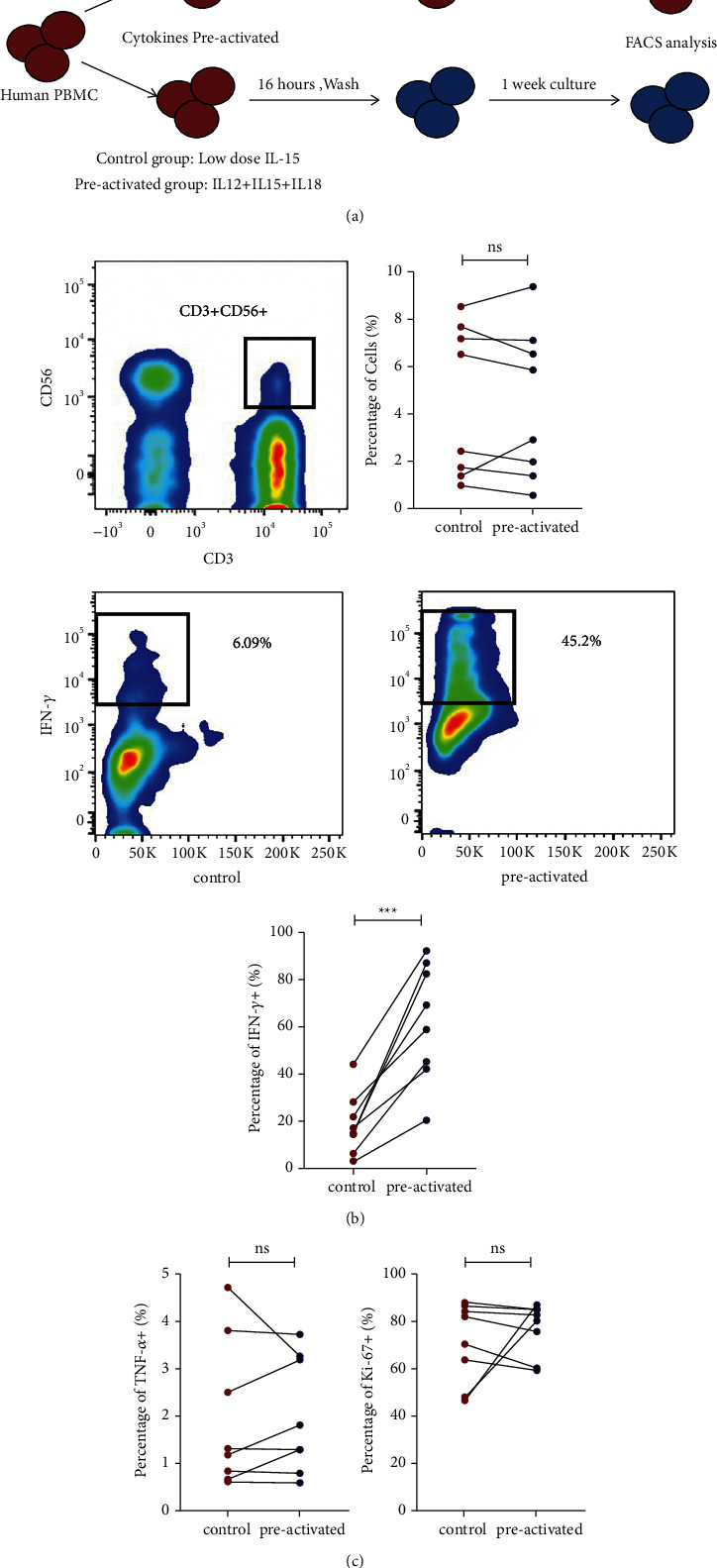
IL-12/15/18-induced CD_3_+CD_56_+ NKT-like cells increase IFN-*γ* release. (a) Overview of experimental design. CD_3_+CD_56_+NKT-like cells were preactivated with rhIL-12 (10 ng/mL), IL-15 (1 ng/ml), IL-18 (50 ng/ml), or control (1 ng/ml of IL-15 alone) for 16 hours. Flow cytometry analysis was performed at indicated time point after preactivation. (b) The gating strategy for CD_3_+CD_56_+NKT-like cells and percentages of IFN-*γ*+ cell populations by flow cytometry plots. Percentages of cells and enhanced IFN-*γ* production restimulated with same IL-12/15/18 on day 7. *n* = 8, none*P* > 0.05,^*∗∗∗*^*P* < 0.001 (error bars, mean ± SEM). (c) Expression of Ki67 restimulated with same IL-12/15/18 on day 7. TNF-*α* production restimulated with same IL-12/15/18 on day 7. *n* = 8, none*P* > 0.05,^*∗∗∗*^*P* < 0.001 (error bars, mean ± SEM).

Human CD_3_+CD_56_+ NKT-like cells were preactivated with low-dose IL-15 as control or with IL-12/15/18 for 16 h, and then the control and experimental samples were cocultured with IL-15 for 7 days to induce training immunity. The cells were analyzed by flow cytometry at different time points (Figure 1(a)). During culture, the proportion of cells did not change (*P*=0.334, Figure 1(b)). Consequently, trained CD3+CD56+ NKT-like cells exhibited enhanced IFN-*γ* production in response to restimulation with IL-12/15/18 for 6 h on day 7 (*P*=0.002, Figure 1(b)), which was similar to the memory effect, but TNF-*α* release and cell proliferation were not improved (*P*=0.126; *P*=0.432, Figure 1(c)).

**Figure 2:**
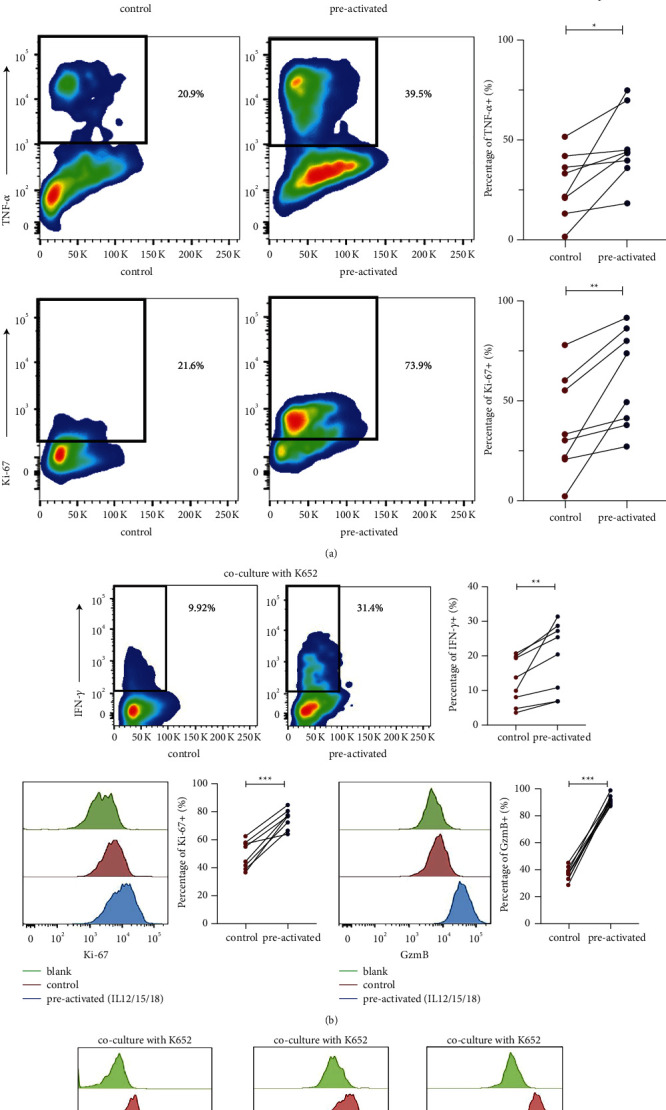
IL-12/15/18 preactivated CD3+CD56+ NKT-like cells enhance the intrinsic potential and show an increase in IFN-*γ* release when stimulated by K562 tumor cells. (a) Percentages of IFN-*γ*+ TNF-*α*+ and Ki67+ cell populations by flow cytometry plots. Expression of IFN-*γ*, TNF-*α,* and Ki67 in cells restimulated on day 7 by cell activation cocktail. n = 8, ^*∗*^*P* > 0.05,^*∗∗*^*P* < 0.01,^*∗∗∗*^*P* < 0.001 (error bars, mean ± SEM).(b) The gating strategy for percentages of IFN-*γ*+ cell populations cocultured with K652 on Day 7; an overlaid histogram shows increased expression of Ki67 and enhanced level of GzmB. *n* = 8. (c) Overlaid histograms show expression of IFN-*γ*, Ki67, and GzmB cocultured with K562 on day 7 by different concentrations of cytokines preactivating.

The preactivated CD_3_+CD_56_+ NKT-like cells showed an increase in IFN-*γ*, TNF-*α*, and cell proliferation potential after PMA + iono stimulation on day 7 (*P* < 0.001; *P*=0.020;*P*=0.008, Figure 2(a)). IFN-*γ* release, granzyme B level, and proliferation ability significantly increased when IL-12/15/18 preactivated CD_3_+CD_56_+ NKT-like cells were stimulated by K562 (*P*=0.013; *P* < 0.001; *P* < 0.001, Figure 2(b)). When the proportion of tumor cells was adjusted, IL-12/15/18 preactivated CD_3_+CD_56_+ NKT-like cells encountered different proportions of K562 tumor cells. The results showed that the effect of different proportions of K562 tumor cells was the same (*P*=0.184; *P*=0.245; *P*=0.245, Figure 2(c)).

### 3.2. Combination of IL-12, IL-15, and IL-18 Can Display the Memory-Like Effect

**Figure 3:**
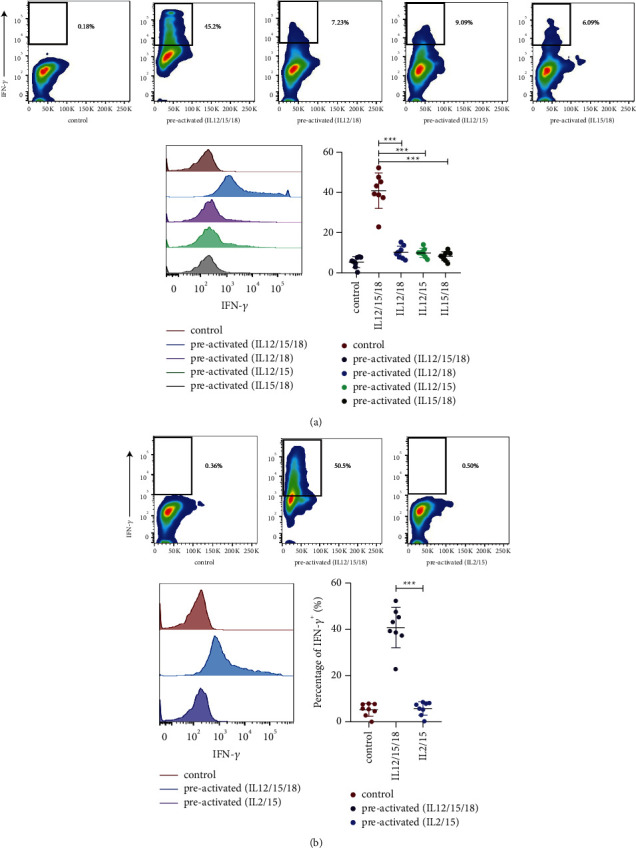
Combination of IL-12, IL-15, and IL-18 can display the memory-like effect. Notes: (a) The gating strategy for percentages of IFN-*γ*+ cell populations restimulated with different combinations of cytokines (IL-12/15/18) on day 7. An overlaid histogram and enhanced IFN-*γ* production by cytokines trained cells restimulated with the same combinations of cytokines on day 7. *n* = 8,^*∗∗∗*^*P* < 0.001 (error bars, mean ± SEM). (b) The gating strategy for percentages of IFN-*γ*+ cell populations restimulated with different combinations of cytokines (IL-2/15) on day 7. Overlaid histograms and IFN-*γ* production by different cytokines trained cells restimulated with the same combinations of cytokines on day 7. n = 8,^*∗∗∗*^*P* < 0.001 (error bars, mean ± SEM).

The experimental group was divided into four groups by using the same experimental method. IL-12/15/18 preactivated CD_3_+CD_56_+ NKT-like cells could produce a memory-like effect, whereas other cytokines, such as IL-12/IL-18, IL-12/IL-15, and IL-15/IL-18, could not stimulate a similar biological effect (*P*=0.934; *P*=0.919; *P*=0.803, Figure 3(a)). Meanwhile, IL-2/IL-15 cytokines could not increase the IFN-*γ* release compared with the cells preactivated with IL-12/15/18 (*P* = 0.805, Figure 3(b)).

**Figure 4:**
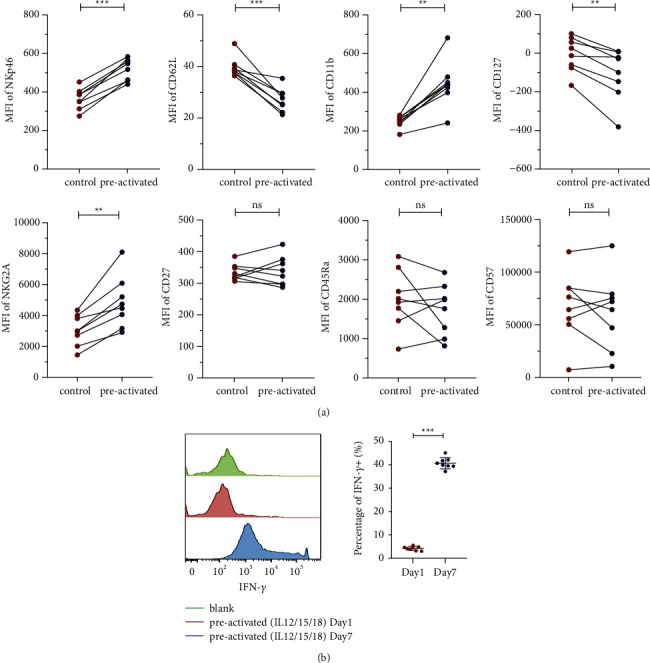
CD11b, NKp46, CD62 L, CD127, and NKG2A of IL-12/15/18 preactivated CD3+ CD56+ NKT-like cells show evident and consistent change trends. (a) MFI shows the expression profile of the cell surface antigens. n = 8. none *P* > 0.05, ^*∗∗*^*P* < 0.01,^*∗∗∗*^*P* < 0.001 (error bars, mean ± SEM). (b) Overlaid histograms and IFN-*γ* production by cytokines trained cells restimulated with the same combinations of cytokines on day 1 and day 7. ^*∗∗∗*^*P* < 0.001(error bars, mean ± SEM).

CD_3_+CD_56_+ NKT-like cells were simulated with cytokines IL-12/15/18 for 16 h and then cocultured with low-dose IL-15 for 7 days. Detecting some differentiation-related surface markers, we found that CD11b, NKp46, CD62 L, CD127, and NKG2A exerted evident and consistent change trends (*P*=0.001; *P* < 0.001; *P* < 0.001; *P* < 0.001; *P* < 0.001, Figure 4(a)). Moreover, the IFN-*γ* release was higher in CD_3_+CD_56_+ NKT-like cells prestimulated by cytokines IL-12/15/18 on day 7 than that prestimulated on day 1 (*P* < 0.001, Figure 4(b)).

**Figure 5:**
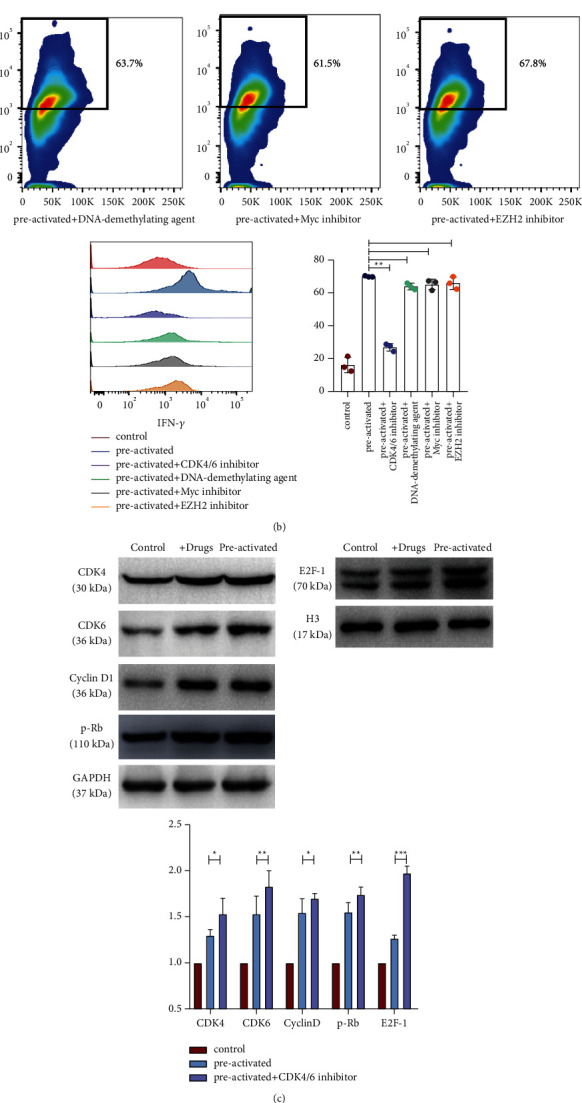
CDK4/6 inhibitors are involved in the formation of cytokine-induced trained CD3+CD56+ NKT-like cells. (a) Overview of experimental design. CD3+CD56+NKT-like cells were preactivated with rhIL-12 (10 ng/mL), IL-15 (1 ng/ml), IL-18 (50 ng/ml) ± CDK4/6 inhibitor (10um/L), or control (1 ng/ml of IL-15 alone) for 16 hours. Flow cytometry analysis was performed at the indicated time point after preactivation. (b) The gating strategy for CD3+CD56+NKT-like cells and percentages of IFN-*γ*+ cell populations; overlaid histograms show repressed expression of IFN-*γ*; percentages of IFN-*γ*+ cell populations declined by preactivated with the CDK4/6 inhibitor/DNA-demethylating agent/Myc inhibitor/EZH2 inhibitor; *n* = 8. (c) Western blot analysis of the expression of CDK4, CDK6, cyclin D1, p-Rb, and E2F-1. ^*∗*^*P* > 0.05, ^*∗∗*^*P* < 0.01,^*∗∗∗*^*P* < 0.001 (error bars, mean ± SEM). (d) The formation of cytokine-trained CD3+CD56+ NKT-like cells，cells stimulated by IL-12/15/18 via the CDK4/6 signaling pathway induce active proliferation and differentiate into trained CD3+CD56+ NKT-like cells with enhanced function molecules expression (IFN-*γ*, TNF-*α*, GzmB, and Ki67).

We selected several small-molecule drugs on the basis of epigenetics to study the mechanism of this memory effect in CD_3_+CD_56_+ NKT-like cells, including the CDK4/6 inhibitor, DNA-demethylating agent, Myc inhibitor, and EZH2. We added these small-molecule drugs to the IL-12/15/18 preactivated CD_3_+CD_56_+ NKT-like cells on day 1 and trained CD_3_+CD_56_+ NKT-like cells using the above-mentioned method (Figure 5(a)). The IFN-*γ* release significantly reduced in the experimental group with the CDK4/6 inhibitor, whereas the IFN-*γ* release in the other three drug experimental groups did not significantly decrease (*P* < 0.001; *P*=0.963; *P*=0.297; *P*=0.158, Figure 5(b)). We detected the expression of CDK4/6 and its downstream pathway proteins by the Western blot to verify whether CDK4/6 and its downstream pathway proteins are involved in the biological effects of CD_3_+CD_56_+ NKT-like cells. The experimental results showed that after adding the CDK4/6 inhibitor, the expression level of CDK4/6 in the drug group was significantly lower than that in the preactivated group (*P*=0.008, Figure 5(c)). The expression of cyclin D1, the phosphorylation level of Rb, and the expression of E2F-1 significantly decreased (*P* < 0.001, Figures 5(c) and 5(d)).

## 4. Discussion

CD_3_+CD_56_+ NKT-like cells are a large group of unique cell subsets that express TCR and NK cell markers, accounting for approximately 5%–15% of the peripheral T cell pool and up to 50% of T cells within the liver [14]. Different from the traditional T cells, CD_3_+CD_56_+ NKT-like cells have innate and adaptive immune functions, T cells, and NK cells [1, 14]. Therefore, they can perform MHC-restricted cytotoxicity or MHC-unrestricted cytotoxicity and secrete a variety of cytokines [1, 2, 5]. CD_3_+CD_56_+ NKT-like cells play an important role in antitumor and antiviral immune response [4]. The activation of CD_3_+CD_56_+ NKT-like cells may be crucial because they can serve as an early source of regulatory cytokines and degranulation-related killing functions [4, 5]. IFN-*γ* is the key factor in activating the acquired immune response and resisting various infections, autoimmune diseases, and tumor infections [1–3, 5]. It primarily exerts antiviral activity and specific cytotoxicity and promotes Th1 transformation through overexpression of MHC class I and II molecules, antigen processing, and immunoglobulin conversion [1, 2, 14]. Therefore, IFN-*γ* may be an important indicator for CD_3_+CD_56_+ NKT-like cells to exert immune function, and it is important to study the relationship and changes among these cells.

Recent studies have found that cytokine signal transduction is important for the differentiation, survival, maintenance, and activation of NK cells [12, 13, 15]. The cytokines IL-2, IL-12, IL-15, IL-18, and IL-21 and type I IFNs have been used to expand and activate NK cells in vitro before adoptive transfer, and this process efficiently boosts the quantity and function of NK cells [15–17]. In the study of memory NK cells, IL-12/15/18 preactivated NK cells showed an increase in IFN-*γ* production in response to restimulation with IL-12/15/18 or K562 tumor cell lines. Compared with traditional NK cells, cytokine-induced memory NK cells had stronger recognizing and killing ability [5, 12, 17, 18]. As an important natural immune cell, CD_3_+CD_56_+ NKT-like cells have many similar characteristics with NK cells in killing activity, such as cytotoxicity of sensitive tumor cells and expression of NK cell receptors to regulate their own functions [1, 14]. Therefore, we deeply explore whether cytokine-induced CD_3_+CD_56_+ NKT-like cells, which are similar to memory NK cells, have biological characteristics to improve their role in resisting infection and tumor. Our study found that IL-12/15/18 preactivated CD_3_+CD_56_+ NKT-like cells exhibited enhanced IFN-*γ* production in response to restimulation with IL-12/15/18 for 6 h on day 7, which was similar to the memory effect, but the TNF-*α* release and cell proliferation were not improved. Among these cytokines, the combination of IL-12/15/18 was effective, whereas other cytokines, such as IL-2/IL-15, IL-12/IL-18, IL-15/IL-18, and IL-12/IL-15, could not stimulate a similar biological effect. Therefore, our study found that CD_3_+CD_56_+ NKT-like cells showed similar memory characteristics after cytokine activation, which may provide a new theoretical basis for CD_3_+CD_56_+ NKT-like cell immunotherapy.

Tumor immunotherapy is a potential therapeutic method [7, 19], which can be used to remove cancerous cells by activating immune cells in the body, and the advantages of tumor immunotherapy include high specificity, long action period, and small side effects [6–8, 19]. Trained immunity is orchestrated by epigenetic reprogramming, broadly defined as sustained changes in gene expression and cell physiology that do not involve permanent genetic changes, such as mutations and recombination, which are essential for adaptive immunity [7, 20]. Therefore, the discovery of trained immunity may lead to new therapeutic strategies for the treatment of many immune diseases and could be a potential strategy for tumor immunotherapy. Some studies have focused on CD_3_+CD_56_+ NKT-like cells in tumor. Jiang YongJun found that the function of CD_3_+CD_56_+ NKT-like cells in long-term nonprogressors may be a protective mechanism to slow down the progression of HIV disease [14]. Peng Liu-Sheng indicated that the frequencies of CD_3_+CD_56_+ NKT-like cells in gastric cancer tumors significantly decrease and that low levels of tumor-infiltrating CD_3_+CD_56_+ NKT-like cells are positively correlated with poor survival and disease progression [1]. In patients with chronic myelogenous leukemia, Jani-Sofia Almeida observed significant alterations on the expression of tumor recognition (NCRsandNKp80) and immune regulatory receptors (LAG-3, TIM-3, and CD137) by NKT-like cells [2]. These studies suggest that CD3+CD56+ NKT-like cells play an important role in the occurrence and development of cancer. In the present study, the preactivated CD3+CD56+ NKT-like cells showed an increase in IFN-*γ*, TNF-*α*, and cell proliferation potential after PMA + iono stimulation on day 7, indicating that training immunization can directly activate the intracellular signaling pathways without being affected by cell surface receptors. When we stimulated these cytokine-induced CD_3_+CD_56_+ NK-T-like cells with K562 tumor cells, we found that the IFN-*γ* release, granzyme B level, and proliferation ability were significantly increased. K562 tumor cells can directly stimulate these trained CD_3_+CD_56_+ NKT-like cells to release IFN-*γ* and enhance their killing ability, which also indicates the potential of cytokine training immunity for clinical tumor therapy. The memory effect of CD_3_+CD_56_+ NKT-like cells induced by cytokines is primarily manifested in the significantly increased release of IFN-*γ* after encountering the same cytokine stimulation. This finding also suggests that after cytokine immune training, CD_3_+CD_56_+ NKT-like cells can mobilize their internal potential and play a stronger immunological function when they encounter appropriate stimulation. Therefore, optimizing the cytokine scheme, inducing CD_3_+CD_56_+ NKT-like cells sensitive to certain tumor cells, and making this immune cell safe, stable, and effective in killing tumor cells in patients with cancer is necessary.

The specific mechanism of the memory effect in cytokine-trained CD_3_+CD_56_+ NKT-like cells remains unclear, and we should consider the research process, causes, and results. In the present study, we compared the CD_3_+CD_56_+ NKT-like cells preactivated with cytokines IL-12/15/18 on the first day (about 16 h) with those maintained for 7 days. When the same dose of IL-12/15/18 was restimulated, the IFN-*γ* release was significantly increased on the 7th day but not on the 1st day, suggesting that the cultured cells must remain stable for a period of time to induce a memory-like effect, which also indicates that their formation exists during cell division and differentiation. In addition, we detected the expression of indicators on the surface of the cell membrane, and the results showed that after preactivation with IL-12/15/18, some cell surface proteins related to function and differentiation, such as CD11b, CD62 L, NKp46, NKG2A, and CD127, showed evident and consistent change trends, suggesting that this memory-like effect manifested in different differentiation states. On the basis of epigenetics, we selected several small-molecule drugs and observed their effects on the experimental results. Cyclin-dependent kinases (CDKs), first discovered by Tim Hunt, Paul Nurse, and Leland H. Hartwell, play an important role in the initiation of cell cycle and the regulation of cell cycle transition [21, 22]. Human cells have a complete division and proliferation cycle, from G1 phase to S phase and G2 phase to M phase [23–25], and CDK4/6 in CDKs are key condition proteins of human cell division and proliferation cycle [23]. CDK4/6 inhibitors selectively inhibit CDK4/6, effectively preventing tumor cells from progressing from G1 phase to S phase, restoring cell cycle control, and blocking tumor cell proliferation [21–23]. Our results showed that when some small-molecule drugs were added to the culture induced by cytokines on day1, the Myc inhibitor, DNA-demethylating agent, and EZH2 could not change the IFN-*γ* release, whereas CDK4/6 inhibitors can effectively weaken this effect, suggesting that this memory-like effect may be related to cell cycle and cell proliferation. Moreover, after adding the CDK4/6 inhibitor, this biological effect has not completely disappeared, suggesting that its production mechanism results from multiple effects, which is still closely related to the cell microenvironment or other unknown proteins and receptors, and more research must be further explored. The Western blot results showed that CDK4/6 and cyclin D could phosphorylate the retinoblastoma gene (Rb) and then release the transcription factor E2F, which could promote the transcription of cell cycle-related genes, thereby regulating the occurrence of this memory-like phenomenon. This phenomenon provides insights into the mechanism study of such memory-like CD_3_+CD_56_+ NKT-like cells.

The present study proposed that CD_3_+CD_56_+ NKT-like cells in PBMCs preactivated by IL-12/15/18 could gain trained immunity with enhanced effector functions, a memory-like phenomenon, analyzing the possible response of these prestimulated cells to K562 tumor cells. In addition, this study found that IFN-*γ* played a key role in the proliferation and cytotoxicity of CD_3_+CD_56_+ NKT-like cells. The important role of CDK4/6 in this memory response was also proposed, which provided experimental reference for other factors of its formation mechanism and paved the way for later research and clinical research. Tumor immunotherapy is a potential therapeutic method in the 21st century [7, 8], which can be used to clear cancer cells by activating immune cells in vivo. Tumor training immunotherapy is modified on the basis of epigenetics. Epigenetics regulates the chromatin status and gene expression through DNA methylation and demethylation, histone modification, and chromatin remodeling, but it does not involve permanent genetic changes, which is important for adaptive immunity [7, 26, 27]. Therefore, our study may provide a new theoretical basis for CD_3_+CD_56_+ NKT-like cells in immunotherapy strategies, but further study on their differentiation, survival, maintenance and activation of microbial homeostasis, and clinical transformation of the idealized model of specific killing tumor cells is necessary.

## 5. Conclusions

Our work revealed that cytokine IL-12/15/18 can induce CD_3_+CD_56_+ NKT-like cells to obtain enhanced training immunity, which was a memory-like phenomenon [28].

## Data Availability

The data and materials used to support the findings of this study are available from the corresponding author upon request.

## Abbreviations

SEM: standard error of the mean
GAPDH: Glyceraldehyde-3- phosphate ehydrogenase
H3: histones 3
GzmB: Granzyme B
ns: No significant.
SEM: standard error of the mean
SEM: standard error of the mean
MFI: median fluorescence intensity
